# Case for diagnosis. Lichen myxedematosus*

**DOI:** 10.1590/abd1806-4841.20165725

**Published:** 2016

**Authors:** Priscila Regina Orso Rebellato, Mauren Beatriz Frazon Carbonar, Nicole Iasmin Magario Tabuti, Graziela Junges Crescente Rastelli

**Affiliations:** 1 Faculdade Evangélica do Paraná (Fepar) – Curitiba (PR), Brazil; 2 Universidade Positivo (UP) – Curitiba (PR), Brazil; 3 Universidade Federal do Paraná (UFPR) – Curitiba (PR), Brazil

**Keywords:** Mucinoses, Paraproteinemia, Scleromyxedema

## Abstract

Scleromyxedema or lichen myxedematosus is a rare papular mucinosis of chronic and progressive course and unknown etiology. It is commonly associated with monoclonal gammopathy and may show extracutaneous manifestations, affecting the heart, lung, kidney, and nerves. The diagnosis is based on four criteria: generalized papular and sclerodermoid lesions; mucin deposition, fibroblast proliferation, and fibrosis in the histopathology; monoclonal gammopathy; and no thyroid disorders. This article reports the case of a scleromyxedema patient with a recent history of acute myocardial infarction and monoclonal gammopathy.

## CASE REPORT

A 51-year-old male presented with multiple papules that had been present for one year. He reported difficulty moving his fingers and pain in his proximal interphalangeal articulation, which worsened with heat. He had suffered an acute myocardial infarction one month before and was on treatment for dyslipidemia.

**Figure 1:**
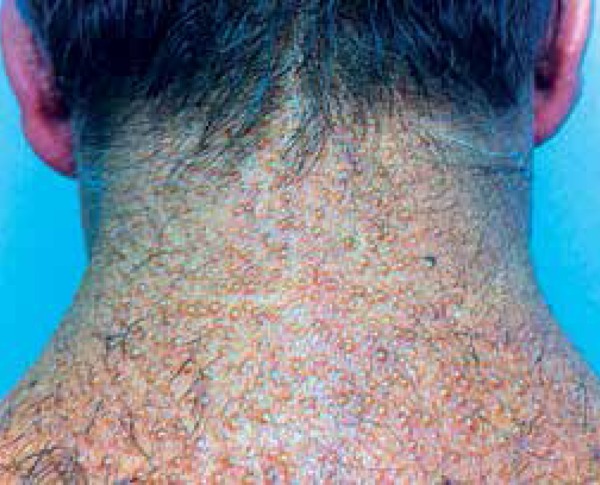
Whitish and normochromic millimetric papules in the posterior cervical region

**Figure 2:**
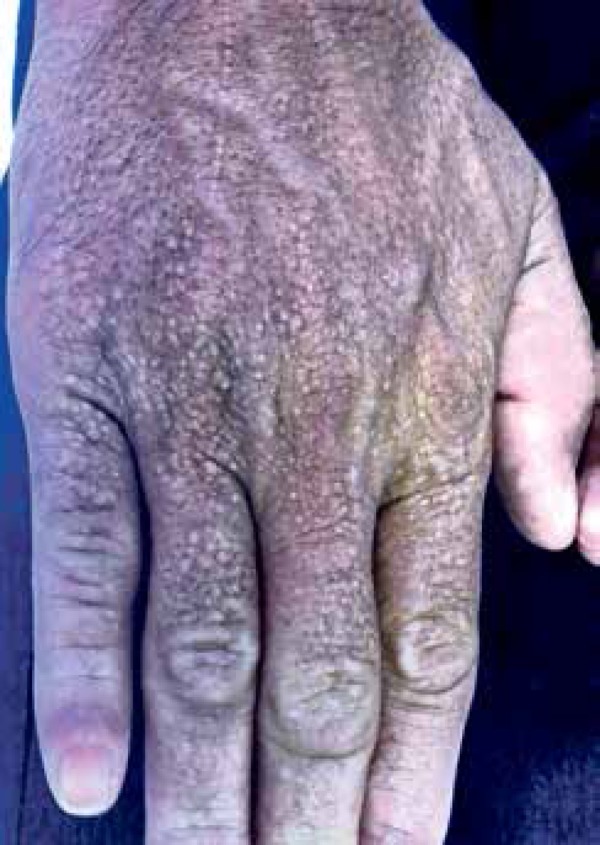
Whitish and normochromic millimetric papules on the back of the right hand

Dermatological examination showed whitish and normochromic millimetric papules on his neck, back, hands, feet, abdomen, and in the inguinal region (Figures 1 and 2). In addition, his face and earlobes showed infiltration.

**Figure 3:**
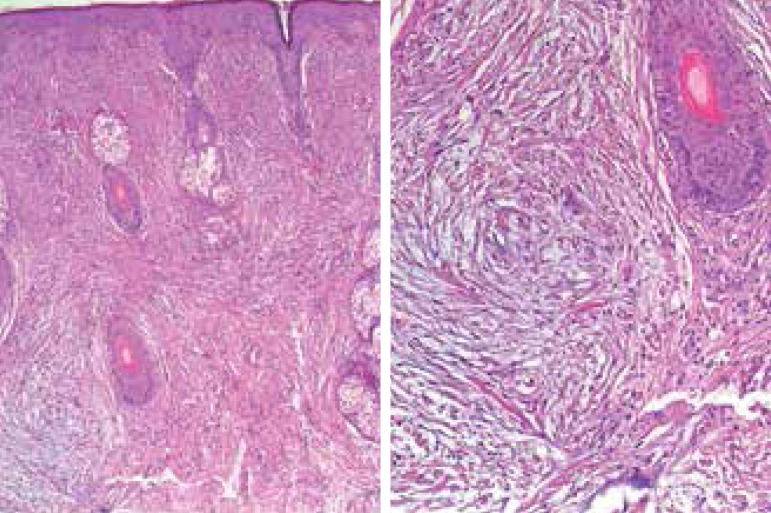
Characteristic triad: fusiform cells (fibroblasts) in the superficial and mid layer of the dermis, fibrosis, and pronounced mucin deposition (HE)

**Figure 4:**
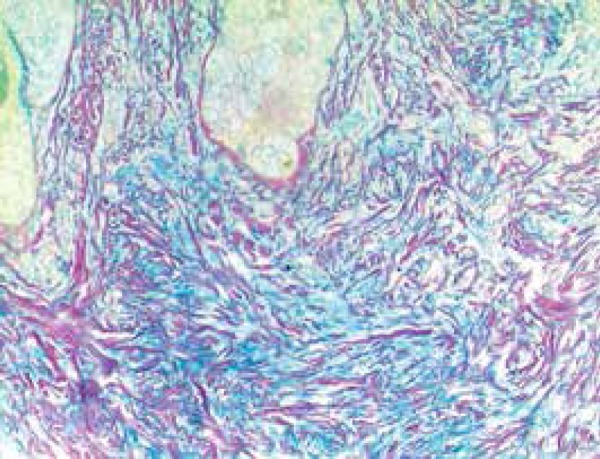
Mucin deposition dissecting the dermal collagen fibers (colloidal iron)

Histopathology found revealed mucin deposits and fibroblast proliferation in the reticular dermis, the hypodermis, and a mild perivascular lymphocytic infiltrate. The epidermis was mildly acanthotic, and the basal layer was normal as well as the hypodermis (Figures 3 and 4).

Protein electrophoresis showed a peak of gamma globulin of 21.8% (11.1% - 18.8%) and of beta-2 microglobulin at 2,095 ng/ml (normal values up to 2,000 ng/ml). Other laboratory tests were unremarkable.

## DISCUSSION

Scleromyxedema or lichen myxedematosus (LM) is a rare type of papular mucinosis, with a chronic and progressive course, chronic, progressive, of unknown etiology. It is commonly associated with monoclonal paraproteinemia^1-3^ and is characterized by papular lesions associated with erythema and thick, diffuse scleroderma-like changes.^2^ The normochromic or erythematous papules are stiff and grouped, ranging from 1 mm to 4 mm. They are symmetrically arranged, primarily on the back of the hands and fingers, the extensor surface of the arms, the face, the upper torso, and the legs. Scalp and mucosae are not affected.^1,4^ They may coalesce, resulting in widespread induration of the skin and eventually leading to leonine facies and microstomia.^1,2^

In some cases, LM may be associated with multiple myeloma, acute leukemia, and T-cell lymphoma.^1^ Extracutaneous manifestations include dermato-neural syndrome, myopathy, inflammatory polyarthritis, esophageal disorders, changes in the larynx and nerves, pulmonary disease, and heart and liver abnormalities.^1,2^ Cardiac abnormalities occur in 10% of cases, characterized by mucin deposition in the middle layer and adventitia of the myocardial vessels, as well as mucinous degeneration of the atheromatous plaques of the arteries.^5^ Association with systemic hypertension has also been reported.^5^

The diagnosis is based on four criteria: generalized papular and sclerodermoid lesions; mucin deposition, fibroblast proliferation, and fibrosis in the histopathology; monoclonal gammopathy; and no thyroid disorders.^6^ The patient showed all four criteria.

Histological exam of the upper dermis shows a horizontal band of mucinous material between the round, stellate-shaped and irregularly distributed collagen fibers and fusiform fibroblasts,^1,2^ as well as dermal fibrosis.^3^

Differential diagnoses include systemic sclerosis, amyloidosis, scleredema, lichen nitidus, drug-related drug-related lichenoid eruptions, scleroderma, disseminated syringoma, pityriasis rubra pilaris, leprosy, and lichen tuberculid.^2,3^

there is no established treatment.^2^ Alkylating agent melphalan was considered a first-line treatment, but limited use due to its side effects.^1-3^

Other treatments described include cyclophosphamide, intralesional infiltration of hyaluronidase and triamcinolone, CO 2 laser, methotrexate, cyclosporine, radiotherapy, thalidomide, plasmapheresis, 2'-deoxyadenosine (2-CD), systemic corticoid, chloroquine, intravenous immunoglobulin, retinoids, chemotherapeutic agents, and PUVA, but results may vary.^1,7^
